# Reviewed article: Pires MCS, Silva MJS, Araújo PP, Retto MPF. Use of medications in women with triple-negative breast cancer between 2018 and 2019 in a Brazilian public hospital: a retrospective study. Epidemiol Serv Saude. 2025:34;e20240180. 10.1590/S2237-96222024v33e20240180.en

**DOI:** 10.1590/S2237-96222025v34e20240180.b

**Published:** 2025-02-21

**Authors:** Sheilla Siedler Tavares

**Affiliations:** 1 Centro Universitário Facens , Sorocaba, SP, Brazil

## Peer review B

## Questionnaire

Does the manuscript contain new and significant information to justify publication? Yes

Does the Abstract (Summary) clearly and accurately describe the content of the article? No

Is the problem significant and concisely stated? Yes

Are the methods described comprehensively? Yes

Are the interpretations and conclusions justified by the results? Yes

Is adequate reference made to other work in the field? Yes

## Manuscript Structure

Length of article is: Adequate

Number of tables is: Adequate

Number of figures is: Adequate

Please state any conflict(s) of interest that you have in relation to the review of this paper (state “none” if this is not applicable).

None

Interest: Good

Quality: Good

Originality: Good

Overall: Good

Recommendation: Minor Revision

## Comments

Sugiro pequenas alterações/reflexões: no título acrescentar arsenal terapêutico medicamentoso.

Nos resultados, sempre que utilizar dados númericos colocar as porcentagens ao lado, exemplo sete (25%), 10 (50%).

Escrever o resumo de maneira mais abrangente nos resultados, respondendo o objetivo.

Curiosidade: essa população deste estudo sofreu alguma consequência da COVID-19? Acham que vale a pena pontuar algo no artigo sobre isso?

